# Anthelmintic resistance of gastrointestinal nematodes in cattle in Brazil and Argentina - current status and global perspectives

**DOI:** 10.1590/S1984-29612024041

**Published:** 2024-08-12

**Authors:** Fernando de Almeida Borges, Alessandro Francisco Talamini do Amarante, Welber Daniel Zaneti Lopes, Candela Canton, Luis Alvarez, Adrian Lifschitz

**Affiliations:** 1 Universidade Federal de Mato Grosso do Sul – UFMS, Campo Grande, MS, Brasil; 2 Universidade Estadual Paulista – UNESP, Botucatu, SP, Brasil; 3 Universidade Federal de Goiás – UFG, Goiânia, GO, Brasil; 4 Laboratorio de Farmacología, Centro de Investigacíon Veterinaria de Tandil – CIVETAN, UNCPBA-CICPBA-CONICET, Facultad de Ciencias Veterinarias, Campus Universitario, Tandil, Argentina

**Keywords:** Cooperia, Haemonchus, livestock management, drug combinations, cattle health, Cooperia, Haemonchus, manejo na pecuária, combinação de drogas, saúde de bovinos

## Abstract

This review outlines the current state of anthelmintic resistance (AHR) of gastrointestinal nematodes (GINs) among cattle in Argentina and Brazil, emphasizing the economic repercussions, animal health and welfare. The analysis explores factors associated with AHR and proposes a potential solution: the use of drug combinations. Both countries are grappling with a severe AHR scenario in cattle, having progressed through incipient, established, and advanced phases, leading to extreme cases of animal mortality due to ineffective control strategies. Genera such as *Cooperia* and *Haemonchus* have the highest reports of resistance, with *Oesophagostomum radiatum* also posing significant problems. While oral benzimidazoles and levamisole remain effective in most herds, moxidectin is entering an advanced resistance phase, and avermectins are increasingly deemed ineffective. The review explores the impact ofclimate, mixed grazing, animal movement and other husbandry practices, and the relationship between ectoparasite control and the emergence of resistant helminths. Notably, the discussion includes the strategic use of drug combinations as a valuable approach to address resistant GINs control in livestock, highlighting its significant potential to mitigate the challenges posed by AHR in the cattle industry of these countries.

## Resumo

Esta revisão tem como objetivo delinear o estado atual da resistência anti-helmíntica (RAH) em nematódeos gastrintestinais (NGIs), em bovinos na Argentina e no Brasil, enfatizando o impacto econômico na saúde e no bem-estar animal. A análise explora fatores associados à RAH e propõe uma solução potencial: o uso de combinações de medicamentos. Ambos os países enfrentam um cenário grave de AHR, progredindo por meio de fases incipientes, estabelecidas e avançadas, levando a casos extremos de mortalidade animal, devido a estratégias de controle ineficazes. Os gêneros *Cooperia* e *Haemonchus* apresentam mais relatos de resistência, enquanto *Oesophagostomum radiatum* também pode causar problemas significativos. Embora os benzimidazóis orais e o levamisol permaneçam eficazes na maioria dos rebanhos, a moxidectina está entrando em uma fase avançada de resistência, e as avermectinas são cada vez mais consideradas ineficazes. A revisão explora o impacto do clima, pastoreio misto, movimentação dos animais, práticas de criação e a relação entre o controle dos ectoparasitas e o surgimento de helmintos resistentes. Notavelmente, o uso estratégico de combinações de medicamentos pode ser uma abordagem valiosa para o controle de NGIs resistentes em bovinos, destacando-se o seu potencial significativo na mitigação dos desafios colocados pela RAH naqueles países.

## Introduction

Investigation of the evolution of anthelmintic resistance (AHR) and the factors related to it in Argentina and Brazil can contribute to knowledge about this serious problem that is already widespread globally in cattle. Together, the two countries account for 63% of the territory, 60% of the population and 62% of the gross domestic product (GDP) of South America, and their integration would give them the sixth largest economy in the world. Although there are some striking differences in cattle farming between these two countries, some common characteristics make it interesting to know their AHR scenario: both countries have been involved in the first reports of AHR in cattle; most publications reveal the predominance of the genera *Cooperia* and *Haemonchus* in field infections; and while a large number of commercial anthelmintic formulations are available, preference is given to the use of injectable endectocides; and past treatment has generally been with a single drug (avermectin). Furthermore, in both countries, cattle farming is a major contributor to GDP and extensive grazing is an important stage of the production system (virtually the only one in Brazil) (Malafaia et al., 2021).

Therefore, livestock farmers in both countries face similar challenges in implementing strategic gastrointestinal nematodes (GINs) control programs and performing routine diagnostic exams. The presence of large herds (>10,000 animals/farm) also limits the use of refugia-based strategies. For this reason, we highlight drug combination as a possibility to manage AHR.

## The AHR Scenario in Brazil and Argentina

### Chronology

The evolution of AHR of GINs in cattle in Brazil and Argentina (Chart 1) can be summarized in three phases: 1) the first reports, with the majority of cases pertaining to *Cooperia* spp. resistant to ivermectin (IVM); 2) emergence and generalization of populations resistant to multiple drugs and participation of *Cooperia*, *Haemonchus*, *Oesophagostomum* and *Trichostrongylus* in cattle after treatments, identified by necropsies or coprocultures (more details and references are presented in Chart 1), period marked by lack of perception of the problem by technicians and farmers, resulting in lower animal performance; and 3) large field surveys indicating a greater number of farms with AHR and lower average efficacy of products, which, combined with other farming factors resulted in a resurgence of outbreaks, with calves exhibiting diarrhea, apathy, bottle jaw, weight loss and death (Carmo et al., 2011; Pivoto et al., 2020b; Lima et al., 2022).

**Chart 1:** Reports of anthelmintic resistance in cattle in Argentina (AR) and Brazil (BR).

Year	Country	Diagnostic Method	Drugs	Species/Genus						Other relevant results	Reference
				C	H	N	Oe	Os	T		
1990	BR	Necropsy	BZDs		X					T, Os and C were susceptible	Pinheiro & Echevarria (1990)
2001	BR	LDA	IVM	X							Paiva et al. (2001)
2001	AR	FECRT and necropsy	IVM, LA-IVM, DRM, MOX	X							Anziani et al. (2001)
2001	AR	FECRT and necropsy	IVM, LA-IVM, DRM,	X						MOX and FBZ: efficacy >95%.	Fiel et al. (2001)
2003	AR	FECRT and necropsy	IVM and BZD	X	X			X			Mejía et al. (2003)
2004	AR	FECRT	IVM and BZD	X	X					LEV: efficacy >95%	Anziani et al. (2004)
2004	AR	FECRT	AVMs	X							Anziani & Fiel (2004)
2005	BR	FECRT	IVM and DRM		X					MOX and ABA: efficacy >99%	Rangel et al. (2005)
2006	BR	FECRT	IVM, ABA, DRM and MOX	X					X	H susceptible to ABA	Mello et al. (2006)
2007	BR	FECRT	IVM, AS and LEV	X	X					25 farms. Percentage of farms with FECRT< 90%: IVM-92%, LEV-20%, AS-8%, MOX-0%	Soutello et al. (2007)
2007	AR	FECRT	IVM and BZD	X				X		25 farms. Percentage of farms with AHR: IVM-60%, FBZ-32%, LEV-0%.	Suarez & Cristel (2007)
2008	BR	FECRT	IVM, AS and LEV	X	X					39 farms. Percentage of farms with AHR: IVM-82.1%, LEV-15.4% and AS-7.8%	Souza et al. (2008)
2008	BR	Necropsy	IVM and DRM	X							Cardoso et al. (2008)
2009	BR	Necropsy	MOX	X			X			H susceptible to MOX	Condi et al. (2009)
2009	BR	Necropsy	IVM and LA-IVM	X	X		X		X	High efficacy of Trichlorfon	Lopes et al. (2009)
2010	BR	FECRT	IVM, LA-IVM, ABA and DRM	X	X			X	X	Os was resistant only to IVM. MOX and SA: efficacy >95%	Cezar et al. (2010)
2010	BR	FECRT	IVM	X						MOX and SA: efficacy >95%	Soutello et al. (2010)
2010	AR	FECRT	IVM and MOX	X							Lifschitz et al. (2010)
2011	BR	FECRT	IVM, LA-IVM, AS, ABA and DRM	X	X					High frequency of treatments (8 treatments/2 years). Dairy cattle.	Costa et al. (2011)
2011	AR	Necropsy	IVM	X		X			X		Fiel et al. (2011)
2012	BR	FECRT	IVM and DRM	N.I.	N.I.	N.I.	N.I.	N.I.	N.I.	ABA and SA: efficacy >95%	Bruhn et al. (2013)
2013	BR	FECRT	IVM, LA-IVM and LA-DRM	X	X						Borges et al. (2013)
2013	BR	LMIT	IVM	X						8 farms. 100% of isolates were resistant.	Almeida et al. (2013)
2014	BR	Necropsy	LA-IVM	X	X		X			6 farms.	Felippelli et al. (2014)
2014	BR	FECRT	IVM, MOX and AS	X	X		X		X	10 farms. Percentage of farms with AHR: IVM-100%, MOX-80%, LEV-10% and AS-10%. Mean efficacy of MOX>80%.	Neves et al. (2014)
2014	BR	Necropsy	IVM	X	X		X				Lopes et al. (2014a)
2014	BR	Necropsy	LA-IVM	X	X					MOX: efficacy >95%	Lopes et al. (2014b)
2014	AR	FECRT	IVM and MOX	X						LEV+RBZ: efficacy = 99%	Fazzio et al. (2014)
2015	BR	Necropsy	IVM, LA-IVM and LA-DRM	X	X		X				Borges et al. (2015)
2015	BR	FECRT	LA-IVM	X	X		X		X		Holsback et al. (2015)
2016	BR	FECRT	IVM, DRM, EPR, FBZ, CLO, NIT, DIS, LEV, AS and MOX	X	X				X	11 farms. Combinations MOX+LEV, DRM+FBZ and LEV+CLO: efficacy>95%.	Ramos et al. (2016)
2016	BR	FECRT	DRM, MOX and LEV	X	X						Heckler et al. (2016)
2017	AR	FECRT	IVM	X	X						Canton et al. (2017)
2017	AR	FECRT	IVM	X	X			X		62 farms. Percentage of farms with AHR (genus): IVM-93.5% (C and H), RBZ-27.9% (C and Os), FBZ-7.4 (H), LEV-0%.	Cristel et al. (2017)
2018	AR	FECRT	IVM	X							Canton et al. (2018)
2020	BR	FECRT	MLs and AS	X	X					LEV: efficacy>95%	Pivoto et al. (2020a)
2020	BR	FECRT	LMs	X	X					A case of clinical helminthosis culminating in the death of steers. LEV and AS: efficacy>95%	Pivoto et al. (2020b)
2020	BR	FECRT	IVM	X	X					BZDs: efficacy>95%	Neves et al. (2020)
2021	BR	FECRT								FBZ: efficacy=100%	Nakatani et al. (2021)
2021	BR	FECRT	IVM	X	X					BZDs: efficacy>95%	Neves et al. (2021)
2021	BR	FECRT	IVM, BZD, CLO	N.I.						20 farms. Percentage of farms with AHR: IVM-95%, AS-95%, CLO-75% and LEV-20%.	Melo et al. (2021)
2022	BR	FECRT	LA-IVM	X	X					A case of clinical helminthosis culminating in the death of cattle.	Lima et al. (2022)
2022	BR	FECRT	IVM, LA-IVM, MOX	X	X						Borges et al. (2022)

ABA = abamectin; AS = albendazole sulphoxide; AVM = avermectin; BZD = benzimidazole; CLO = closantel; DIS = disophenol; DRM = DRMmectin; LA-DRM = long-acting doramectin; EPR = eprinomectin; FBZ = fenbendazole; IVM = ivermectin; LA-IVM = long-acting ivermectin; LEV = levamisole; MOX = moxidectin; NIT = nitroxinil; FECRT = Fecal egg reduction test; LDA = Larval development assay; LMIT = Larval migration inhibition test; N.I. = not informed; C = *Cooperia*; H = *Haemonchus*; N = *Nematodirus*; Oe = *Oesophagostomum*; Os = *Ostertagia*; T = *Trichostrongylus*.

**Figure 1:**
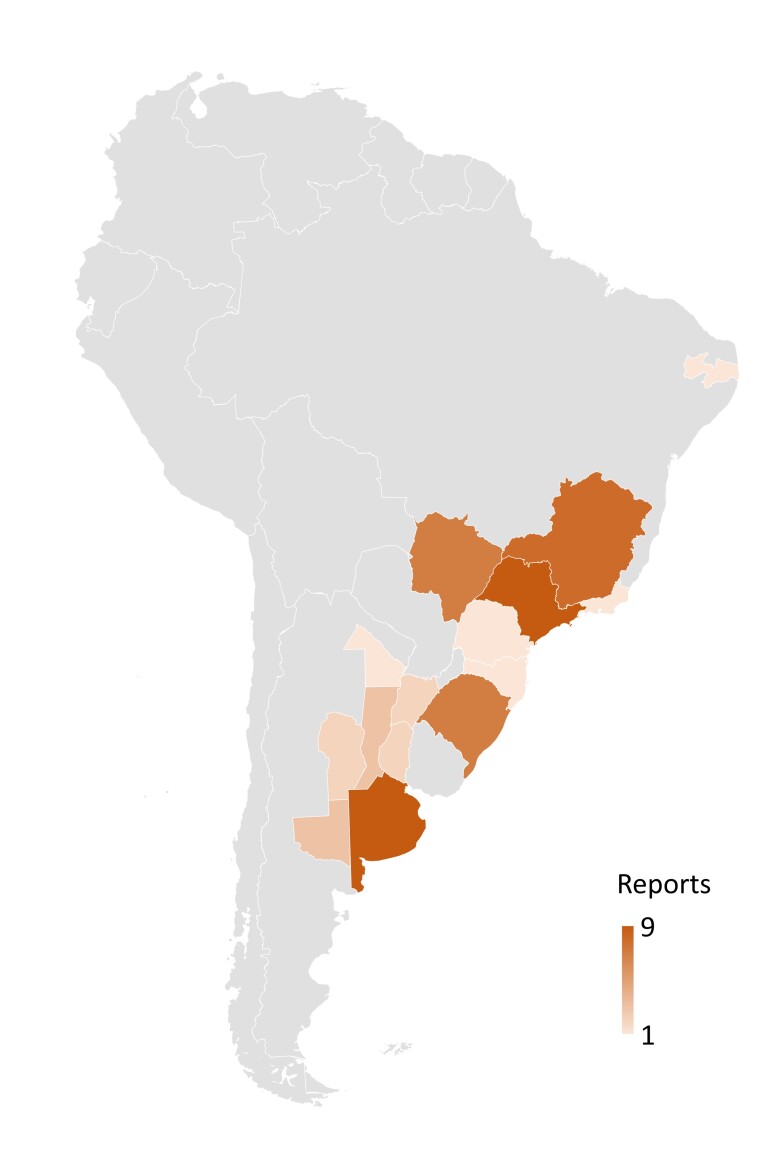
Number of reports of anthelmintic resistance in gastrointestinal nematodes of cattle according to location in Argentina (provinces) and Brazil (states).

Currently, the AHR scenario in Brazil and Argentina can be considered one of the most serious in the world. Since in 2020 in Europe, including UK, Italy and France, the countries with the highest number of reports, had six scientific publications (Vineer et al., 2020), while in Brazil and Argentina there are 33 and 11, respectively (Figure 1), in addition to cases of multi drug resistance and multiple species (Chart 1). Indeed, there are now no longer any reports of farms without GINs resistant to at least one drug in these two countries.

### AHR severity in Argentina and Brazil

Based on the results of fecal egg count reduction tests (FECRTs) and controlled anthelmintic tests carried out in both countries (Chart 1), the GIN resistance scenario in cattle to different drugs can classified as avermectins in a serious situation, moxidectin (MOX) and subcutaneous benzimidazoles (BZDs) in an alert situation, and oral BZDs and levamisole (LEV) still effective.

#### AVMs

Since the first reports in 2001 (Anziani et al., 2001; Fiel et al., 2001; Paiva et al., 2001), the resistance of *Cooperia* spp., and to a lesser extent other genera, to IVM has been demonstrated in tests on just one farm or in large surveys (Chart 1), ranging from 60% to 100% of farms (Soutello et al., 2007; Suarez & Cristel, 2007; Souza et al., 2008; Almeida et al., 2013; Felippelli et al., 2014; Neves et al., 2014; Ramos et al., 2016). Currently, these percentages on farms in both countries are very high, with 93.5% in Argentina (Cristel et al., 2017) and 95% in Brazil (Melo et al., 2021).

There are differences in potency between LMs against resistant and susceptible isolates, with MOX and abamectin (ABA) being the most potent, while eprinomectin, doramectin (DRM) and IVM are the least potent (Kotze et al., 2014). This characteristic can explain the generalized resistance to DRM (Chart 1) and some situations in which abamectin (ABA) was effective against some IVM-resistant populations (Rangel et al., 2005; Bruhn et al., 2013). However, in the same period, Cezar et al. (2010) did not observe efficacy of ABA against a multi-resistant isolate. Since this drug has not been included in efficacy studies, the current resistance status is unknown.

#### MOX and subcutaneous BZDs

Although they belong to the group of macrocyclic lactones, differences in the chemical structure between AVM and MOX molecules result in different phenotypic responses of the free-living nematode *Caenorhabditis elegans* and the interaction with Glutamate-gated chloride channels, a site of action of these drugs (Ardelli et al., 2009) and with different levels of gene expression and affinity of the various P-glycoproteins, involved in one of the possible mechanisms of resistance to MLs (Xu et al., 1998; Lespine et al., 2007), resulting in greater MOX potency (Kotze et al., 2014). Therefore, moxidectin may still effective (>95%) against IVM-resistant populations, as observed in seven studies carried out in Argentina and Brazil (Fiel et al., 2001; Rangel et al., 2005; Soutello et al., 2007; Cezar et al., 2010; Almeida et al., 2013; Neves et al., 2014, Lopes et al., 2014b).

Although there are reports of populations of GINs resistant to MOX (Chart 1), the level of resistance is within an efficacy range between 70 and 95%, which can still result in significant improvement in the productive performance of cattle (Borges et al., 2013). This effect was observed in calves in a feedlot in Argentina, where the efficacy of IVM was 28% and that of MOX was 85%, and there was an additional weight gain of 160g/day in calves treated with MOX compared to IVM (Fazzio et al., 2014). A similar result was observed for beef cattle raised in pasture in Brazil (Borges et al., 2022). Despite the possibility of better performance of animals treated with anthelmintics with efficacy below 95%, it is necessary to consider the risk of accelerating the selection process of resistant GINs if they continue to be used (Barnes et al., 1995).

Injectable benzimidazoles also have a resistance profile that can be considered intermediate. Despite several reports of GINs resistant to this drug, there are still IVM-resistant populations that are susceptible to albendazole (ABZ) sulfoxide (Soutello et al., 2007; Cezar et al., 2010; Bruhn et al., 2013; Pivoto et al., 2020b; Neves et al., 2020). There are some reports in which the resistance to injectable BZDs is as serious as that of IVM, such as in the state of Paraíba, Brazil, where 95% of farms had GINs resistant to both drugs (Neves et al., 2021). On the other hand, some studies have shown lower occurrence of resistance to ABZ (7.8 to 27.9% of farms) than to IVM (93.5% to 100% of farms) (Souza et al., 2008; Neves et al., 2014; Cristel et al., 2017).

#### LEV and oral BZDs

If there is a need to recommend the treatment of cattle with an anthelmintic without knowledge of the history of treatments or results of efficacy tests, the drugs of choice would be LEV or oral BZDs, since there are few reports of resistance to them, even in multi-drug resistant GIN populations (see Chart 1).

The low level of resistance to benzimidazoles in cattle in Brazil was confirmed by a broad search for three benzimidazole resistance-associated single nucleotide polymorphisms (SNPs) in the β-tubulin gene in samples of *Haemonchus* spp. In all Brazilian geographic regions, in which frequencies of resistance-associated alleles above background (≥ 15%) were found for at least one codon in 11.4% of the fields (Fávero et al., 2020).

## Consequences of AHR on Health and Productivity

Based on the increase of helminth populations resistant to chemical products, the emergence of health and production problems in cattle is increasingly problematic. In the best-case scenario, while *Cooperia* spp. prevail in small intestine (SI), the animals have reduced genetic potential, mainly affecting the productivity of the herd. In a study in Argentina conducted by Fazzio et al. (2014), the authors showed that the failure of anthelmintic treatment, especially due to the ineffectiveness of the formulation used, can cause the loss of up to 10 kg per animal. And in a study in Brazil conducted by Borges et al. (2013), the authors observed that the use of anthelmintic formulations with efficacies ≤ 50% (3.15% IVM, 3.5% DRM and 1% DRM) did not increase performance. An increase in the productivity of cattle treated with anthelmintics tends to occur when the formulations demonstrate efficacy against helminths of ≥85% (Borges et al., 2015). Similar results were found in New Zealand, where the ineffective control of resistant *Cooperia oncophora* resulted in a 14-kg difference in live weight gain in beef calves at 12 months of age (Sutherland & Leathwick, 2011), and in the U.S.A. (Stromberg et al., 2012) *C. punctata* caused a deleterious effect on dry feed uptake (0.68 kg/day) and weight gain (0.11 kg/day) in a period of 60 days in beef cattle.

In the field, the health and productive damage triggered by the greater abundance of *Cooperia* spp. can be mitigated, even in conditions where resistance occurs, as long as animals are supplemented with protein, as observed by Neves et al. (2020) with untreated animals (weaned male crossbreeds) obtaining similar weight gain to a group treated monthly with IVER (classified as ineffective) and a group of animals treated monthly with ABZ (classified as effective). In a study conducted by Zapa et al. (2021) with weaned Nelore females, not treated with anthelmintics and supplemented with protein, the authors observed that animals with FEC of 0 gained 0.51 kg/day; females with FEC between 300-487.5 gained 0.70 kg/day; and females with FEC between 500 and 1700 gained 0.99 kg/day. One hypothesis to explain these results is the fact that the genetically most productive animals are also the most susceptible to GIN infection. Hence, when the most susceptible animals are supplemented with protein, part of the protein is used to combat helminths, and another for the animal to reach its genetic productivity potential, thus presenting better weight gain compared to animals more resistant to GIN infection (Neves et al., 2020; Zapa et al., 2021).

In more serious situations, in the face of a more advanced AHR selection, there may be a change in the proportion of SI and abomasum (AB) nematodes, with AB parasites becoming prevalent in cattle (*Haemonchus* and *Trichostrongylus*), including in adult animals. This scenario represents a threat for cattle production, since in addition to the drop in productivity, there can be deaths of cattle triggered by these species of GINs (Lima et al., 2022). Recent studies point out some risk factors for the resurgence of clinical cases and even mortality due to GIN infection: (i) flaws in the parasite control protocol, with the use of ineffective products (Lima et al., 2022); (ii) prolonged periods without anthelmintic treatment of animals (Teixeira et al., 2021; Trindade et al., 2023); and (iii) climatic factors that can trigger a shortage of pasture and affect the immunological response of animals during this period (Lima et al., 2022). They also found that failure of the parasite control protocol, by using ineffective products, was responsible for the mortality of cattle, including adult cows, due to high parasitism by *Haemonchus* spp. and *Trichostrongylus* spp. Other studies have demonstrated that 120 to 180 days without the use of anthelmintics caused the proportion of *Haemonchus* spp. to increase in AB, and consequently the proportion of *Cooperia* spp. to decrease in SI (Teixeira et al., 2021; Trindade et al., 2023). In this way, it is possible for animals to show clinical signs such as submandibular edema, weight loss and mortality (Lima et al., 2022). In other words, the presence of clinical signs of GIN infection caused by AB nematodes in cattle tends to occur later, which can probably lead to the death of the animal if no effective therapeutic intervention occurs. In such situations, there will be health and productive damage to the herd, with possible death of animals. This situation raised an alert for technicians and veterinarians to pay attention to this resistance theme, with the aim of reducing health problems and production losses triggered by cattle helminths.

## Anthelmintic Use and Risk Factors for Resistance of Different Nematode Species

### Influence of climate on AHR development

The climate has a major influence on the distribution of different nematode species. *Haemonchus placei, Cooperia punctata* and *Oesophagostomum radiatum* are widespread in Tropical and Subtropical areas of Brazil and Argentina, where they are the leading cause of economic losses to cattle farmers. These are also the major species implicated in cases of AHR in those areas (Lopes et al., 2014a; Neves et al., 2014; Borges et al., 2015). In addition, there are also reports of *Cooperia pectinata*, *Trichostrongylus* spp. and *Trichuris* spp. with AHR in cattle (Souza et al., 2008; Condi et al. 2009; Felippelli et al., 2014).

In the Santa Catarina Plateau, which is one of the regions with the lowest average temperature in Brazil, the presence of *Ostertagia* with resistance to LEV in cattle has also been recorded (Souza et al., 2008). Likewise, in the western Pampeana Region of Argentina, there are reports of AHR involving *O. ostertagi* and *C. oncophora* (Suarez & Cristel, 2007). However, in some areas in Argentina and southern Brazil, with high temperatures in summer and cold in winter, there is coexistence of species typical of tropical climates with those typical of temperate climates, with reports of the presence of a great diversity of species with AHR at the same site. In Argentina, Mejía et al. (2003) reported BZD resistance in the same herd involving *H. placei, O. ostertagi, C. oncophora* and *C. punctata*. Likewise, Anziani et al. (2004) recorded multiple resistance of *H. placei, C. oncophora* and *C. pectinata* to BZD and IVM.

During long periods of dry weather, most of the nematode population is found in the host. However, the application of anthelmintics during this period, when the helminth population in the environment (*in refugia*) is low, might favor the selection of resistant parasites. For this reason, AHR is also widespread in cattle raised in this type of environment (Melo et al., 2021). Likewise, the emergence of resistant parasite populations is favored when animals are treated before being introduced into “clean” pastures. In this situation, only the parasites that survived the treatment (the resistant strains), produce descendants that contaminate the new area. This problem occurred in Argentina when treated herds were transferred to clean areas, previously used for crop production (Suarez & Cristel, 2014). The management system adopted on a cattle farm in Rio Grande do Sul, where newly treated animals were transferred to clean pastures, was also identified as the cause of the serious development of resistance to macrocyclic lactones (Mello et al., 2006).

Due to differences in climatic conditions, in Argentina large regional variations are observed in the frequency of use of anthelmintics. In marginal semiarid regions, with extensive grazing systems, calves are treated only once or twice a year. In the subtropical deforested area or plains, treatments are more frequent (3 to 5 times a year) and aim to control not only endoparasites, but also ectoparasites, with the use of endectocides. In the temperate plains of the southern cone, where mixed grain and cattle production systems predominate, management is much more intensive and drugs are used more frequently, from 4 to 9 times a year (Suarez, 2002). Date of treatment and frequency of treatment in the past with a single drug (mostly IVM) are the main risk factors involved in AHR development in Argentina. High frequency of anthelmintic application as the principal risk factor; especially when associated with the autumn-winter drenching, frequently used along with Nov-Jan drenching and the approach involving treatment before shifting to annual crops. The latter two factors reduce the number of larvae in refugia and increase the selection pressure for resistant GINs (Suarez & Cristel, 2014).

### Mixed grazing of cattle and sheep

During the process of evolution, some GIN species become specialized in parasitizing a restricted zoological group of hosts, giving rise to species with high host-specificity, as is the case of *Oesophagostomum* spp. When cattle and sheep share pastures, *Oesophagostomum* cross-infection is rarely observed: *O. radiatum* infection is restricted to cattle and *O. columbianum* infection to sheep (Santiago et al., 1975). On the other hand, some species, such as *Trichostrongylus axei*, are generalists (low host-specificity), being able to infect both monogastric animals (horses) and ruminants (deer, sheep and cattle) (Santiago et al., 1975; Eysker et al., 1983; Silva et al., 1994; Nascimento et al., 2000). Therefore, in a hypothetical situation where sheep and cattle share the same pasture, if sheep are frequently treated with LEV, it is to be expected that over time the population of *T. axei* will become resistant. Since the same population of *T. axei* also infects cattle, the effectiveness of LEV should also decrease in these hosts. On the other hand, it is unlikely that the same will happen with *Oesophagostomum* species, which have high host-specificity.

Young cattle are more susceptible to GIN infections, so they are more readily infected with some ovine-adapted nematodes. In Germany, calves acquired patent gastrointestinal nematode infections by species that are primarily ovine parasites (*H. contortus, C. curticei, N. battus, T. colubriformis, Chabertia ovina*) while grazing at a sheep-dominated farm (Rehbein et al., 2022). In a beef cow/calf system with the cows and calves grazing in the same pastures as sheep in New Zealand, *H. contortus* was frequently found infecting pre-weaned beef calves. Nevertheless, there was no evidence of an impact of *Haemonchus* alone, or mixed nematode infection, on pre-weaned calf growth rates on these farms (Waghorn et al., 2022). In South Australia, both *H. contortus* and *H. placei* were found infecting cattle (Jabbar et al., 2014).

### Animal movement and husbandry

Resistant parasites can be introduced along with newly acquired animals in a cattle farm. The ability of gastrointestinal nematode strains, translocated by animal movement, to develop and thrive in a new region will depend on successful competition and mating with the locally established parasite population. If there is compatibility between the original population and the introduced population, crossing is expected, giving rise to new genotypes (Sargison et al., 2019). Experimentally, Redman et al. (2012) reported the successful introgression of IVM resistance genes from two independent IVM-resistant strains, MHco4 (WRS) and MHco10(CAVR), into the susceptible genome reference strain MHco3(ISE) using a backcrossing approach.

### Relationship between ectoparasite control and emergence of resistant helminths

The influence of ectoparasite control on the emergence of resistant helminths is a controversial topic. The use of endectocides to control ectoparasites, especially ticks (*Rhipicephalus microplus*), botflies (*Dermatobia hominis*) and screwworms (*Cochliomyia hominivorax*), can indirectly favor the selection of helminths resistant to macrocyclic lactones, especially in European cattle breeds, which are more susceptible to ectoparasites than zebu cattle. In these breeds, macrocyclic lactones used in association with acaricides and/or insecticides (up to 16 treatments per year) in order to control ectoparasites might have aggravated nematode resistance (Molento & Brandão, 2022). Similarly, along the subtropical deforested area or plains of Argentina, frequent treatments with persistent drugs (3 to 5 times per year) are used with the aim of controlling both internal and external parasites (Suarez, 2002). In 20 properties in the semiarid region of Paraíba, with a predominance of dairy cattle, IVM followed by DRM were the antiparasitics used most, not only to control helminths but also to control *R. microplus* (Melo et al., 2021). In that study, resistance to IVM was detected in 19 of the 20 farms.

### Choice of anthelmintics and drenching frequency

In recent decades, macrocyclic lactones have dominated the antiparasitic market for cattle treatment. The emergence of several commercial brands, associated with the low price of the product, has resulted in the intensive and indiscriminate use of endectocides in cattle herds, which has resulted in greater resistance of helminths. In addition to the low price of endectocides, their preferential and massive use is also encouraged by the persistent effect, activity against ectoparasites and ease of administration (Suarez, 2002; Rangel et al., 2005).

Based on data available from the Brazilian National Union of the Animal Health Products Industry, in December 2014 there were 131 commercial avermectin formulations available in Brazil for cattle, with 41 companies selling 77 formulations containing IVM and 34 companies selling 54 products containing ABA (reviewed by Rath et al., 2016). This may explain why resistance to IVM is more common than resistance to ABZ and LEV (Soutello et al., 2007; Neves et al., 2014). Nevertheless, before the launch of IVM in the market, benzimidazoles and imidathiazoles were the anthelmintics most widely used for the strategic control of gastrointestinal nematodes in cattle, dating to the 1970s and 1980s, respectively, in Mato Grosso (Melo & Bianchin, 1977) and Rio Grande do Sul (Pinheiro, 1983).

AHR to LEV was absent in all 61 farms evaluated in seven provinces of Argentina where resistance to IVM and BZDs are widespread. The high efficacy of this drug in Argentina is presumably linked to its infrequent use on cattle (Cristel et al., 2017).

The person responsible for choosing the product and determining the frequency of application of the anthelmintic in the herd is generally the farm owner. Thus, the control of helminths is empirical, as demonstrated in a study carried out in the highlands of Santa Catarina involving 39 properties. In that region, Souza et al. (2008) reported that most owners did not have accurate information about the frequency of treatments and the anthelmintics used. The antiparasitic choice generally depended on price, advertising and vendor’s recommendations. This explains the wide variation in the frequency of application of anthelmintics, not only in Santa Catarina, but also in other Brazilian states. For example, in São Paulo, Neves et al. (2014) reported a range of 2-12 annual treatments (mode = 6) in 10 evaluated farms; Soutello et al. (2007) reported from one to four treatments (mode = 2) in 25 properties, in some cases where two treatments were administered at the same time as the application of the foot-and-mouth vaccine. Among these 25 farms, the most severe cases of AHR (multiple resistance to ABZ, LEV and IVM) occurred on the two farms with the highest frequency of anthelmintic treatments (four times per year). Conversely, on a farm that used a single yearly treatment, anthelmintic efficacy was high: 100% for LEV, ABZ and MOX; and 92% for IVM, in both cases according to the FECRT (Soutello et al., 2007).

### Differences from other countries

In areas with a temperate climate *O. ostertagi* and *C. oncophora* are the most relevant gastrointestinal nematodes (Hildreth & McKenzie, 2020), and they are considered the main species involved in cases of AHR in cattle (Waghorn et al., 2006; Edmonds et al., 2010; Kelleher et al., 2020; Mauger et al., 2022). The situation is similar to that recorded in Brazil and Argentina. Resistance to IVM was detected in all 16 properties evaluated in Ireland, and in some of them resistance to FBZ, LEV and MOX was also detected (Kelleher et al., 2020). Similarly, in an extensive grazing system in southwest Australia, a strong level of AHR was observed on 11 farms, with at least one class of anthelmintic failing to achieve a 95% reduction of fecal egg counts of one or more gastrointestinal nematode species. *Cooperia oncophora* displayed resistance to DRM in 91% of the farms and *Ostertagia* displayed resistance to LEV in 80% of the farms. Fenbendazole resistance was present in both *C. oncophora* and *Ostertagia* in 64% and 70% of the farms, respectively (Mauger et al., 2022). In a random sample of beef cattle herds in North Island of New Zealand, resistance to IVM was noted in 56/61 (92%) farms, to ABZ in 47/62 (76%) farms, and to both IVM and ABZ in 45/61 (74%) farms. The parasites most prevalent in resistant populations were *Cooperia* spp. Resistance of *Ostertagia* spp. to IVM was detected in 4/45 (9%) farms, to ABZ in 15/46 (35%) farms, and to LEV in 4/46 (9%) (Waghorn et al., 2006). According to Sauermann et al. (2024), since 2006, little has changed in cattle farming systems in New Zealand except for the widespread use of LEV to control *Cooperia* spp. in young cattle, leading to the emergence of resistance to LEV in *Cooperia* spp. and *Ostertagia* spp..

## Combined Anthelmintic Treatments to Optimize Parasite Control in Ruminants

The utilization of multiple drugs in treatment regimens to enhance clinical outcomes has numerous advantages over monotherapy. Consequently, combination therapies have become increasingly prevalent to manage complex human diseases such as cancer, HIV, and severe bacterial/fungal infections (Gilad et al., 2021; Shyr et al., 2021). This paradigm shift has propelled the transition from the traditional “one-drug/one-target” approach to the more intricate “multiple drugs/multiple targets” strategy (Cheng et al., 2019). The combination of different drugs can yield a broader spectrum of effects, encompassing heightened drug toxicity, and synergistic or additive effects (van Hasselt et al., 2019). Consequently, a comprehensive exploration of pharmacological research is essential to gain insights into the advantages and potential drawbacks of combined drug treatments (Lanusse et al., 2018).

Due to the intensive and often indiscriminate use of anthelmintics, the sustainability of the drug administration approach to control parasitic diseases in ruminants is under serious threat, primarily due to the rapid spread of drug-resistant parasite populations (Kaplan & Vidyashankar, 2012). In light of this challenge, the concept of combining two or more anthelmintics from distinct chemical families has emerged as a strategic approach to combat AHR. The use of combinations can delay resistance emergence or control parasite populations with existing resistance (Geary et el., 2012). The rationale behind this pharmacological strategy lies in the fact that individual nematodes have a diminished likelihood of developing resistance to multiple drugs with varying mechanisms of action compared to when a singular molecule is administered. Consequently, through combined treatment, fewer resistant nematodes will survive and the resulting population will be diluted with susceptible parasites in the pasture (Bartram et al., 2012). Mathematical models have convincingly demonstrated that resistance inevitably evolves at a slower rate when combined treatments are employed compared to an annual drug rotation, regardless of any fitness cost associated with resistance (Leathwick, 2013). Various conditions have been proposed as relevant for the success of combined treatments. These include the presence of refugia in the pasture and the high efficacy (near 100%) of each molecule present in the combined treatment (Bartram et al., 2012). Nevertheless, model simulation shows that an active ingredient with reduced efficacy against one or more nematode species is still likely to be more effective at slowing resistance when used in combination than when used alone (Leathwick, 2013). The increasing cases of AHR in sheep and cattle have stimulated the development and approval of combinations with two or more anthelmintics in Australia, New Zealand and several Latin America countries. An important discussion centers on the advantages and disadvantages of pre-formulated, fixed-dose commercial preparations containing multiple active ingredients in comparison with the simultaneous administration of distinct anthelmintic formulations using individual drugs. While fixed combinations might unintentionally promote the overutilization of commercial products, the co-administration of separate formulations requires rigorous veterinary supervision to guarantee precise dosing (Geary et al., 2012).

The use of anthelmintic combinations has been amply explored in sheep. The positive impact of combining LEV and fenbendazole was initially assessed in lambs infected with nematodes displaying resistance to a broad spectrum of anthelmintics (Anderson et al., 1988). This therapeutic advantage was further substantiated by utilizing a combination of IVM and LEV in field conditions over a span of three years, where the efficacy of the combined treatment was greater than the single administration. Notably, the implementation of a combination treatment, coupled with a strategy involving 10% untreated lambs, resulted in a significant delay in the development of resistance. This highlighted the advantage of implementing combined treatment integrated with refugia-based strategies (Leathwick et al., 2012). However, triple combinations containing IVM-ABZ-LEV and ABA-oxfendazole-LEV failed to control abomasal parasites such as *Teladorsagia* spp. and *Haemonchus* spp. (Wrigley et al., 2006; Suarez et al., 2014) and intestinal parasites such as *Trichostrongylus colubriformis* (Hodgson & Mulvaney, 2017). Interestingly a pharmacokinetic (PK) interaction was shown of IVM-ABZ-LEV after the combined treatment in sheep. The absorption of LEV increased, the ABZ sulphoxide plasma concentrations decreased and the IVM systemic availability was enhanced with the triple combination (Suarez et al., 2014). In contrast, the combined administration of closantel + MXD in sheep did not markedly alter their disposition kinetics. At 13 days post-treatment, the administration of both molecules as a single active principle reached efficacy levels ranging between 80% and 92%, while the combined oral and subcutaneous (SC) treatments reached 99% efficacy. In this case, the combined effect of closantel + MXD successfully restored the maximum efficacy levels, which were not reached by the individual active ingredients (Suarez et al., 2023).

There are only a few reports of the evaluation of anthelmintic combinations in grazing cattle. Some preliminary results indicate that the combination of injectable macrocyclic lactones (ML) and oral LEV was highly effective in minimizing the transport of ML-surviving parasites between different areas of the USA (Smith, 2014). Under different susceptible and resistance scenarios in Argentina, Canton et al. (2017. 2018) assessed the co-administration of ricobendazole (an injectable BZD) plus IVM and ricobendazole plus LEV. In both studies, no adverse PK interactions were observed after the combined treatments, demonstrating that the co-administration of both anthelmintics did not modify the plasma PK behavior of either drug in cattle. In an IVM-resistance scenario, the combination of ricobendazole and IVM was the only treatment that achieved 100% efficacy against resistant *Haemonchus* spp. Also, the combined treatment accounted for the lowest excretion of eggs to the pasture compared to the single drug administration (Canton et al., 2017). Similarly, high efficacy and production benefits for cattle parasitized with resistant nematodes have been obtained with the combined use of ML (DRM) and BZ (ABZ) (Edmonds et al., 2018). Recently, the administration of anthelmintic combinations was evaluated in 10 beef cattle farms in Australia. Whereas AHR to macrocyclic lactones was detected in 9 farms, with resistance to *Cooperia* and *Haemonchus* spp. in 9 farms, the combination of macrocyclic lactones with LEV or with LEV plus oxfendazole resulted in efficacies of 99-100% (Allworth et al., 2023).

In Argentina, the use of anthelmintic combinations in beef cattle farms was evaluated under different scenarios. In the case of using a combination in a context of a susceptible scenario, the co-administration of ricobendazole plus LEV obtained 100% of fecal egg reduction against all gastrointestinal nematode genera in winter and 96% in spring, due to the increment of *Ostertagia* spp. in the parasite population (Canton et al., 2018). These findings highlight the relevance of knowing the epidemiology of the different gastrointestinal nematode genera in naturally infected calves and the advantages of using nematocidal combinations before significant resistance is developed. Therefore, considering the advantages of slowing the development of resistance to a new drug class likely to be gained by releasing it in combination with one or more of the older anthelmintic classes, Canton et al. (2023) reported that the use of monepantel in combination with BZDs compounds could be a good strategy to extend its lifespan for use in cattle as well as to reverse its poor activity against *Oesophagostomum* spp. Furthermore, Ramos et al. (2016) demonstrated that the use of combinations enables effective control of nematodes in the presence even of multiple drug resistance, where the most effective combinations against multi-resistant nematodes were MXD plus LEV, DRM plus fenbendazole and LEV plus closantel in four farms of the south of Brazil. Similarly, in Argentina effective parasite control was reported after using an injectable combination of IVM + ricobendazole in a multiple-resistant field scenario (Canton, 2018).

Only limited research has focused on assessing the sustainability of combinations as a tool to control gastrointestinal nematodes. A study spanning five years and seven sheep farms evaluated the impact of a parasite management program adhering to best practices on the susceptibility of nematodes to LEV, ABZ, and IVM (Leathwick et al., 2015). The main features of this program were the use of effective anthelmintics, mainly in the form of combinations, and the maintenance of refugia to preserve unselected genotypes. At the beginning of the study, most of the farms had *Teladorsagia* spp. with multiple resistance to the drugs under evaluation. Although in some farms the efficacy levels were very low at the start of program, there was a significant improvement in the effectiveness of LEV and IVM, with some reversion towards susceptibility after five years (Leathwick et al., 2015). Importantly, the combinations were integrated into a broader resistance management strategy rather than being employed in isolation. Another approach to evaluate the sustainability of the combined anthelmintic treatments was the use of the moxidectin-LEV combination during four years at a sheep farm with parasites resistant to IVM and LEV (Luque et al., 2021). The initial efficacy test showed high efficacy for the combined treatment (between 84.3 and 100%) against all parasite genera. After four years of parasite control based on the use of the combined treatment (with a total of 12 anthelmintic treatments), the observed final efficacy (2018) showed no considerable changes, except for *T. colubriformis*, which decreased from 97.4 to 58%.

The gathered evidence concerning the utilization of combinations as a strategic approach for resistant nematode control in livestock has significant value. However, it is crucial to recognize that combinations are not a panacea. Sustaining high levels of efficacy over time is likely contingent upon combined management practices that mitigate the need for treatments and/or alleviate selection pressure. Similarly, using a new active ingredient in combination cannot be regarded as the only solution to prevent the development of resistance. This effort should be in balance with diagnostics, the preservation of drug-susceptible nematode populations within refugia and with the design of sustainable parasite control strategies on an individual farm basis.

## Conclusions and Perspectives

The AHR scenario in cattle in Argentina and Brazil has evolved to an advanced phase, with the possibility of re-emergence of clinical cases and animal mortality. The lack of awareness among livestock farmers regarding this serious problem perpetuates the risk factors for AHR. Generalist solutions can be hard to apply in the field, so the diagnosis and monitoring of AHR along with practical recommendations, such as the use of drug combinations, must be designed individually for each herd.
